# Comparative Study of Two Methods of Enteric Virus Detection and Enteric Virus Relationship with Bacterial Indicator in Poyang Lake, Jiangxi, China

**DOI:** 10.3390/ijerph16183384

**Published:** 2019-09-12

**Authors:** Xiaotong Wen, Huilie Zheng, Fang Yuan, Hui Zhu, Duyi Kuang, Zhiqiang Shen, Yuanan Lu, Zhaokang Yuan

**Affiliations:** 1 School of Public Health, Nanchang University, Nanchang, Jiangxi Province Key Laboratory of Preventive Medicine, Nanchang 330006, China; 406530517824@email.ncu.edu.cn (X.W.); zhenghuilie@ncu.edu.cn (H.Z.); ncuzhuhui@126.com (H.Z.); 2 Office of Public Health Studies, University of Hawaii at Mānoa, Honolulu, HI 96822, USA; fangy@hawaii.edu (F.Y.); tina_kuang@berkeley.edu (D.K.); 3 Tianjin Institute of Health and Environmental Medicine, Tianjin Key Laboratory of Risk Assessment and Control Technology for Environment and Food Safety, Tianjin 300050, China; szq922990@126.com

**Keywords:** enteric virus, indicator, water quality, nucleic acid detection method

## Abstract

Currently, water contaminated with fecal matter poses a threat to public health and safety. Thus, enteric viruses are tested for as a part of water quality indicator assays; however, enteric viruses have not yet been listed in the criteria. Effective and sensitive methods for detecting enteric viruses are required in order to increase water safety. This study utilized enteric viruses as possible alternative indicators of water quality to examine fresh water in six sites in Poyang Lake, Nanchang, Jiangxi Province. The presence of norovirus geno-groups II (NoV GII), enteroviruses (EoV) and adenoviruses (AdV) were determined using Tianjin’s protocol and Hawaii’s protocol during a six month period from 2016–2017. The former used an electropositive material method for viral concentration and Taqman-q reverse transcription polymerase chain reaction (RT-PCR) to detect enteric viruses; while the latter used a filtration-based method for viral concentration and RT-PCR for enteric virus detection. There is a statistically significant difference between Tianjin’s method and Hawaii’s method for the detection of enteric viruses, such as NoV GII, EoV, and AdV (*n* = 36, *p* < 0.001). The enteric viruses showed no significant positive correlation with bacteria indicators (*n* = 36, *p* > 0.05). These data stress the need for additional indicators when establishing water quality systems, and the possibility of using enteric viruses as water quality indicators. It has become essential to improve shortcomings in order to search for an adequate method to detect enteric viruses in water and to implement such method in water quality monitoring.

## 1. Introduction

Recently, the increasing morbidity due to waterborne diseases (such as paralysis, gastrointestinal disease and dermatosis) caused by enteric virus has led to public concern worldwide [1,2]. The deteriorating water environment has provided a nurturing basis for enteric viruses. The presence of pathogenic enteric viruses poses a great danger to human health [3,4,5]. Millions of people die from waterborne diseases every year all over the world [6,7]. Typhiod, dysentery, cholera, gastrointestinal diseases and other waterborne diseases occur frequently in China [8,9,10]. A wide variety of enteric bacteria and viruses have been associated with waterborne gastroenteritis [11,12]. They can cause bodily harm through drinking, inhalation, consumption, and direct skin contact with water [1,11,12].

Currently, the safety of water bodies is established through the microbiological examination of the water samples. In China, water quality criteria rely entirely on the use of indicator microbes such as total coliform (TC), fecal coliform (FC), aerobic bacterial count (ACC) and *Escherichia coli* (*E. coli*) to evaluate potential pollution in water. Whereas in the USA, water quality criteria rely on the use of fecal indicator bacteria (FIB) such as *E. coli* and *Enterococcus* (ENT) to assess the risk of waterborne diseases. However, these microorganisms reportedly have deficiencies as indicator microorganisms, as several studies have shown that these bacteria indicators are not always associated with the incidences of human diseases caused by viruses, including gastrointestinal and respiratory diseases [13,14,15]. In addition, indicator bacteria can survive and reproduce in water involving environments other than human and animal feces. This results in an inaccurate estimation of the risk of human illness related to fecal contamination [16,17]. Human enteric viruses have high survival ability in aquatic environments even in extremely low concentrations, these viruses can survive in water for several months [15]. At present, the methods for detecting enteric virus in water mainly contain cell culture technique and polymerase chain reaction (PCR), the traditional cell culture technique method detect the infectivity of enteric virus by observing cell lesion, which is labor-intensive and time-consuming [18]. PCR method is practiced gradually by investigators that based on the constantly developing nucleic acid diagnostic technique with its advantage of high specificity and sensitivity [18].

The Poyang Lake area, where the study was carried out, has been classified as the largest freshwater lake in China with a large water accumulation in the downstream of Yangtze River [19]. However, with an increasing population and a developing economy, the dilemma of ecological environmental destruction and water pollution is becoming increasingly serious issues. In order to decrease the associated risk to the population, it is advised to further assess water quality. This study used two different methods of viral concentration and detection to assess the water quality in Poyang Lake. One method was established in Tianjin Institute of Health and Environmental Medicine and uses electropositive material method for water concentration and real-time fluorescence quantitative PCR for viral detection. The other method was established at the University of Hawaii and uses a filtration-based method and reverse transcription polymerase chain reaction (RT-PCR). 

The goals of this study were to (1) examine the presence of bacteria and enteric viruses in Poyang Lake water during different periods of time and different sampling sites, (2) compare the relative merits of the two aforementioned methods of viral detection, provide reference for choosing appropriate and reliable method for enteric virus detection and related waterborne illness diagnostics and (3) examine the correlation between indicator bacteria concentration and enteric virus presence.

## 2. Materials and Methods 

### 2.1. Description of Sites 

**Figure 1:**
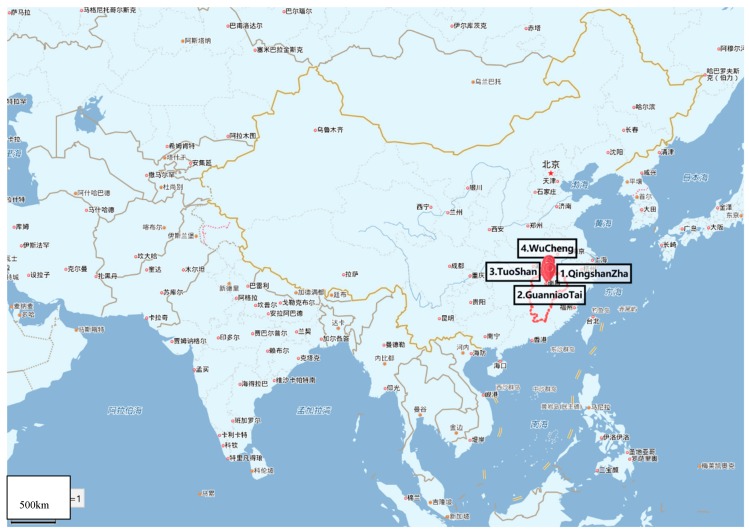
Distribution of sampling sites in China.

**Figure 2:**
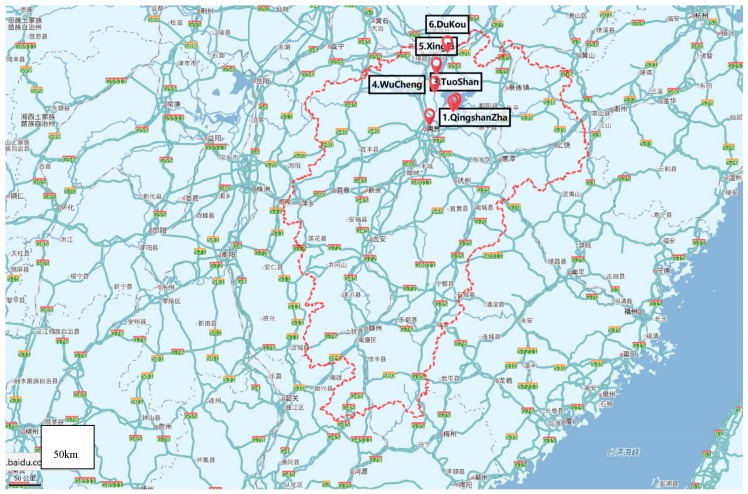
Distribution of sampling sites in Jiangxi Province.

**Figure 3:**
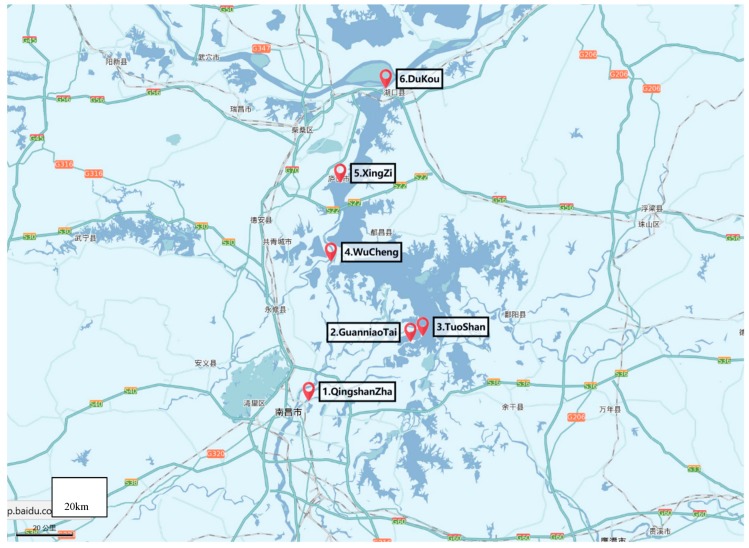
Distribution of sampling sites in Poyang lake.

Poyang Lake is the largest freshwater lake in China located in northern Jiangxi Province, and flows through the middle of the province to the Yangtze River (Figure 1 and Figure 2). Poyang Lake is connected with five main rivers including the Gan River, Hu River, Xin River, Rao River, and Xiu River. Due to its surroundings and natural resources, Poyang Lake provides local residents with the opportunity of economic recreation through fishing and processing a large amount of drinking water. Therefore, the water quality of Poyang Lake is a non-negligible serious public health concern. In this study, water samples were taken from six locations along the Poyang Lake considering factors such as human sources and environmental impact. The site Qingshanzha located in the Gan River was selected as it is an important domestic water outlet from the urban area in Nanchang. Guanniaotai and Tuoshan belong to Nanjishan Water of the southwest of Poyang Lake and were selected because of their biological resources especially because of the migrating birds that may excrete feces into water. The Xingzi and Wucheng sites were selected for their citizens who live near Poyang Lake and visitors. Lastly, the Dukou site, downstream of Poyang Lake that flows into Yangtze River which may be affected by upper river water quality. The site descriptions are summarized in Table 1 and Figure 3. The latitude and longitude of the sites sampled were measured by GPS and recorded. The temperature of Poyang lake water at these sites were measured every sampling time at each site. 

### 2.2. Water Sampling

Six sites were sampled at seven different time periods between May of 2016 and January of 2017. Four time points (October, November, December and January) corresponding to the dry season, and two time points (May and June) corresponding to the rainy/wet season. Since the heavy rainfall may cause the inaccuracy of detection in Poyang Lake in July and August, for this reason, we did not collect water samples during those two months. In total, thirty-six water samples were collected from six sites along the Poyang Lake, in Nanchang, Jiangxi Province. 10-L water samples were collected using sterilized 10-L polypropylene containers for the method established by the University of Hawaii (named Hawaii’s method) [20,21], and 50-L water samples were collecting using sterilized 25-L polypropylene containers for the method established by Tianjin Key Laboratory of Risk Assessment and Control Technology for Environment and Food Safety (named Tianjin’s method). These water samples were transported on ice to the laboratory of Nanchang University and immediately processed for viral detection. 

### 2.3. Bacteriological Water Analyses

Bacteriological analyses of ACC, TC and FC were carried out according to the filter membrane method of GB/T 5750.12-2006 Standard Examination Methods for Drinking Water. The bacteriological culture media used were nutrient agar, endo agar, and MFC according to instruction for ACC, TC and FC respectively. Briefly, 1 mL of different dilution water was passed through 0.45 µm pore size, 39 mm diameter millipore membrane filters. After filtration, the media plates were incubated at 36 °C for 48 h for ACC, at 36 °C 18–24 h for TC, and 44.5 °C for 24 h for FC.

### 2.4. Detection of Enteric Viruses

#### 2.4.1. Viral Concentration by the Electropositive Material Method, Nucleic acid Extraction and Taqman-q RT-PCR Detection (Tianjin’s Method)

Distilled water was added in the mounted filter column for about 30 cm, then 800 g pre-prepared positively charged filter material were slowly added in the filter column while stirring. The filter material is the positively charged filter media particle which is made by the Tianjin Institute of Health and Environmental Medicine. Briefly, following the slow addition of 12.6 g AlCl_3_ to 1890 mL of distilled water slowly at room temperature, 90 mL of a 4 mol/L of Na_2_CO_3_ was then slowly added into AlCl_3_ solution to form an Al(OH)_3_ precipitate which was adjusted to pH 7.2 with Na_2_CO_3_. The solution was further mixed for 15 min and additional Na_2_CO_3_ was added, as required, to maintain a pH of 7.2. The solution was centrifuged at 1100 g for 15 min and the supernatant was discarded. The resulting sediment was resuspended in 1 L 0.14 mol/L NaCl and recentrifuged at 1100 g for 15 min. The supernatant was again discarded and the sediment was resuspended in 1L of 0.14 mol/L NaCl, and then sterilized by autoclaving. The solution was cooled to room temperature, centrifuged again at 1100 g for 15 min and the supernatant was discarded. The Al(OH)_3_ sediment was resuspended in 2 L of sterile 0.14 mol/L NaCl. Next, 340–400 g of silica gel with a 60−100 mesh size (Kunhai Co., Shanghai, China) was added to 1L of the Al (OH)_3_ precipitate, mixed well and dried at 60 °C for 36 h [22,23]. The 50-L water sample was passed through the filter column with the speed of filtration was controlled at 0.5 L/min. Next, 2.4 L glycine complex triple nutrient broth was used for each water sample elution. Glycine complex triple nutrient broth is composited by NaOH (20 g/L), NaCl (15 g/L), anhydrous Na_2_CO_3_ (0.3 g/L), tryptone (30 g/L), beef powder (15 g/L) and glycine (37.5 g/L). As well, PEG6000 was added for adsorption until the final concentration reached 13%. The eluted solution above was centrifuged at 10,000 rpm/min at 4 °C for 30 min for the second concentration, supernatant removed while stroke-physiological saline solution was applied to wash the tube wall to 40 mL [24]. The 40 mL solution was immediately used for experiment or stored at −80 °C for a week. The total concentration process lasted for at least 6 h.

Ribonucleaic Acids (RNA) were extracted from 140 μL eluent using QIAamp Viral RNA Mini Kit (Qiagen Company, Germany). This specific method was done strictly according to the Kit introduction instructions. Deoxyribonucleic acid (DNA) was extracted from 1 mL eluent using UNIQ-10 Order Viral Genome DNA Extraction Kit (Biotech Corporation, Shanghai, China). This specific method was done strictly according to the kit introduction instructions. 

Reverse transcription was first performed using a cDNA first-strand synthesis system (Thermo Fisher Scientific, Waltham, MA, USA) for PV-1. Six microliters of RNA were added to the 14 µL reaction mixture, which contained 4 µL of 5X reverse transcription buffer, 25 units of RevertAid M-MuLV Reverse Transcriptase, 2 µL of 25X deoxynucleoside triphosphates, 10 units of RiboLock RNase inhibitor and 1 µL of each antisense primer (10 µmol/L). The RT reaction mixture was sequentially incubated for 5 min at 65 °C, 60 min at 42 °C and 5 min at 70 °C in a 2720 thermocycler (Applied Biosystems, USA) to synthesize cDNA, and then held at 4 °C until qPCR amplification was performed. qPCR was performed using Platinum PCR SuperMix-UDG (Invitrogen, Carlsbad, CA, USA) in an ABI sequence detection system 7300 (Applied Biosystems, Foster City, CA, USA) (Table 2). A total volume of 20 µL was used for qPCR, including 2 µL of DNA from HAdV or cDNA from NoV II or Eov, 10 µL PCR SuperMix-UDG, 0.5 µL of each 10 µmol/L primer, and 0.5 µL of 5 µmol/L TaqMan probe. The reaction mixture was heated at 50 °C for 2 min and then 95 °C for 2 min. It was followed by a two-step cycling protocol which consisted of a denaturation step at 95 °C for 15 s, and an annealing/extension step at 60 °C for 30 s over 40 cycles. Virus detection was conducted using real-time fluorescence quantitative PCR (RT-qPCR) and tested for the three viruses: NoV GII, EoV, and AdV. 

#### 2.4.2. Sample Concentration by the Filtration-Based method, Nucleic Acid Extraction and RT-PCR Detection (Hawaii’s Method)

Water samples were concentrated using a previously described filtration-based method established at the University of Hawaii [25,26,27]. Five mL of 5 mol/L MgCl_2_ was added to each one liter of water sample. After waiting for 5 min to enhance viral adsorption, the samples were concentrated using 0.45 μm pore size type HA negatively charged filtered membranes (EMD Millipore, Billerica, MA, USA) under vacuum filtration using two sets of sterile forceps. The white filter membrane was picked up at opposite edges and rolled into a cylinder with the top side facing inward. One liter of water was filtered for each freshwater sample; the filtration process lasted about one hour. The filter was inserted into the 5 mL PowerWater^®^ Bead Tube (MoBio Laboratories, Inc., Carlsbad, CA, USA). 

Nucleic acids were extracted from the recovered membranes using the Power Water^®^ RNA Isolation Kit (MoBio Laboratories, Inc., Carlsbad, CA, USA) with the instruction for designed extraction of both DNA and RNA [28,29].

- Preparatory work before starting:
  1. The temperature of the water bath pot is set at 55 °C;
  2. Solution PWR1 must be warmed at 55 °C for 5–10 min to dissolve precipitates prior to use. Solution PWR1 should be used while still warm.
- Designed extraction of both DNA and RNA:
  1. Add 990 µL of PWR1 and 10 µL of βME directly to the PowerWater^®^ Bead Tube. Make sure cap is securely tightened on tube. Vortex at maximum speed for 5 min. Centrifuge the tubes 4000 rpm 4 °C for 1 min at room temperature. Transfer all the supernatant to a clean 2 mL Collection Tube (provided). Draw up the supernatant using a 1 mL pipette tip by placing it down into the beads. Centrifuge at 4000 rpm 4 °C for 1 min. Avoiding the pellet, transfer the supernatant to a clean 2 mL Collection Tube (provided);
  2. Add 200 µL of Solution PWR2 and vortex briefly to mix. Incubate at 4 °C for 5 min. Centrifuge the tubes at 13,000 rpm 4 °C for 1 min. Avoiding the pellet, transfer the supernatant to a clean 2 mL Collection Tube (provided);
  3. Add 650 µL of Solution PWR3 and 650 µL of Solution PWR4. Then vortex briefly to mix. Load 650 µL of supernatant onto a Spin Filter and centrifuge at 13,000 rpm 4 °C for 1 min. Discard the flow through and repeat until all the supernatant has been loaded onto the Spin Filter;
  4. Shake to mix Solution PWR5 before use. Add 650 µL of Solution PWR5 and centrifuge at 13,000 rpm 4 °C for 1 min. Discard the flow through and centrifuge again at 13,000 rpm 4 °C for 1 min to remove residual wash. Place the Spin Filter basket into a clean 2 mL Collection Tube (provided);
  5. Add 650 µL of Solution PWR4 and vortex briefly to mix. Centrifuge the tubes at 13,000 rpm 4 °C for 1 min. Avoiding the pellet, transfer the supernatant to a clean 2 mL Collection Tube (provided);
  6. Add 62 µL of Solution PWR8 (don’t shake) and incubate at 4 °C for 5 min. Centrifuge at 13,000 rpm 4 °C for 1 min. Discard the Spin Filter basket;
  7. A total of 60 µL of nucleic acids were extracted from each sample; 45 µL was used for RNA extraction and 15 µL was used for DNA extraction. 45 µL was treated with 5 µL of PWR6 and 4 µL of DNase I for removal of residual DNA. Incubate at room temperature for 20 min and heating for 5 min for RNA extraction prepared. RNA extraction should be used immediately or stored at −20 °C within 48 h to avoid degradation. The remaining 15 µL was stored at −80°C for viral DNA assays.

Reverse transcriptase was performed using the Easy Script^®^ One-Step gDNA Removal and cDNA Synthesis Super Mix (Transgene, China).

1. Add 1 µL of Random Primer and 8 µL of sample RNA. Vortex briefly to mix and be warmed at 65 °C for 5 min, then ice bath for 2 min.
2. Add 10 µL of 2 × ES Reaction Mix and 1 µL of Easy Script^®^ RT/RI Enzyme Mix to every reaction system. The total volume of every reaction system is 20 µL.
3. Reaction conditions is 25 °C for 10 min, 42 °C for 30 min and 85 °C for 5 min, then be used immediately for experiment or stored at −20 °C for a week.

Then cDNA samples were subjected to qualitative PCR protocols established previously, and tested for three viruses: NoV GII, EoV, and AdV. The first PCR product was used as template for the second amplification under the same PCR condition. PCR reaction conditions and primers set for each virus are summarized in Table 3 and Table 4. PCR mix containing were able to generate PCR products of the expected sizes in a 25 μL volume reaction containing 1X Taq reaction buffer (Mg^2+^ free) (New England Biolabs, NEB, Ipswich, MA, USA), 1.5 mM MgCl2 solution (NEB, Ipswich, MA, USA), 200 nM of each dNTP (Sigma-Aldrich, St. Louis, MO, USA), 0.1 μg/μL of BSA (NEB, Sequence (5’^®^ 3’)a +/– b, Ipswich, MA, USA), 400 nM of forward and reverse primers (Integrated DNA technologies, Coralville, IA, USA), and 2 units of Taq DNA polymerase (provided by Dr. Tung Hoang, University of Hawaii at Manoa), with a Master Cycler Gradient (Eppendorf, Germany). Amplification started with an initial denaturation at 94 °C for 5 min, followed by 40 cycles of denaturation at 94 °C for 30 s, annealing at 56 °C for 30 s, extension at 72 °C for 30 s, and a final extension at 72 °C for 5 min.

Results were visualized using gel electrophoresis on a 2% agarose gel stained with ethidium bromide and examined under ultraviolet light. 10 mL PCR product + 2 mL 6x loading dye was loaded into the wells of an ethidium-bromide stained 2% agarose gel in 0.5x TBE buffer, to which 120 V was applied until sufficient fragment migration had occurred. Then several positive second PCR products were selected and sent to the Beijing Genomics Institute (BGI) for two directional sequencing. The sequencing data was jointed and compared to the all available nucleic acid sequences in the National Center for Biotechnology Information (NCBI) database using Basic Local Alignment Search Tool (BLAST).

### 2.5. Statistical Analyses

Statistical analyses were carried out using SPSS version 24.0 (IBM Corporation, Chicago, IL, USA,). Difference in detection of two methods were analyzed using McNemar Test. Log10-transformed values were used for the computation and test of ACC, TC, FC and *E. coli.* One-sample K-S normal distribution hypothesis test was employed in order to determine the distribution of those bacteria indicators. The Spearman rank correlation was used to test the relationship between bacterial indicators and enteric viruses. A *p*-value below 0.05 was considered statistically significant, and all *p*-values were given as two-tailed. 

## 3. Results

### 3.1. The Distribution of Bacteria Indicators

ACC concentrations were measured at all six sites. While Dukou site demonstrates the highest concentrations compared to others, at site Qingshanzha, TC and *E. coli* concentrations were also relatively higher than other sites. ACC count and fecal pollution indicator bacteria (FIB) such as TC and FC all met bacterial level standards (Table 5). The result of the K-S hypothesis testing shows that the distribution of bacteria indicators all satisfy the Log-normal distribution (*p* > 0.05). 

### 3.2. Detection of Enteric Viruses through Hawaii’s Method Qualitative PCR 

Six sites of Poyang Lake all detected norovirus geno-groups II (NoV GII), enterovirus (EoV), and adenovirus (AdV) through Hawaii’s method. The result of the detection of enteric viruses such as NoV GII, EoV and AdV through Hawaii’s method did not show difference among different various seasons, diffenent flood seasons and different sampling site (*p* > 0.05). The detection rate of adenovirus was the highest (66.67%), and the detection rate of enterovirus and norovirus II was 61.11% and 36.11% respectively, as shown in Table 6.

### 3.3. Detection of Enteric Viruses through Tianjin’s Method Quantitative PCR

All six sites of Poyang Lake all detected NoV GII, EoV and AdV through Tianjin’s method (Table 7). The amount of NoV GII was was detected to be the the highest (6154197581 GC/L) at Dukou site in November of 2016, that of EoV was detected to be the highest (8003342 GC/L) at Tuoshan site in December of 2016, and that of AdV was detected to be the highest (8793346 GC/L) at Xingzi site in January of 2017. The detection rate of AdV was the highest (91.67%), and the detection rate of EoV and NoV GII was 86.11% and 77.78% respectively, as shown in Table 8. 

### 3.4. Comparisons of the Results of the Two Methods by McNemar Test

Water samples which are from the same sites at the same time points were measured for enteric viruses through Tianjin’s method and Hawaii’s method. Use McNemar Test to compare the detection of enteric viruses through Tianjin’s method and Hawaii’s method. There is statistically signifiant difference between Tianjin’s method and Hawaii’s method for the detection of enteric viruses such as NoV GII, EoV and AdV (*p* < 0.05). The positive rate of detection of enteric viruses by Tianjin’s method higher than Hawaii’s method (Table 9). 

### 3.5. Comparisons by Two Methods

The initial volume of eluent for nucleic acid that the Tianjin method employed was used respectively for DNA: 1 ML, and RNA: 140 µL; the method utilized by the University of Hawaii was precisely 600 µL for both DNA and RNA. However, the final volume of nucleic acid was different, with a total of 60 µL in Hawaii’s protocol, and a total of 180 µL of nucleic acid in Tianjin’s protocol in which provides enough templates for quantitative testing. From the perspective of the operations’ time consumption, Hawaii’s protocol spends less than or equal to one hour, which largely saves time in the process of viral concentration and extracting nucleic acid, while Tianjin’s protocol spends at least six hours, mainly on the process of viral concentration (Table 10). 

### 3.6. Correlation of Enteric Viruses between Bacteriological Index in Poyang Lake

Six sites were sampled to analyze both bacteria counts and viral presence. As shown in Table 11, the enteric viruses showed no significant positive correlation with bacteria indicators (*p* > 0.05). The spearman’s correlation coefficients between the enteric viruses and bacteria indicators all are low.

## 4. Discussion 

Use of indicator bacteria to assess the hygienic quality of water is a widely debated issue [33]. Previous studies found that bacteria indicators were inadequate for predicting the occurrence of viruses in water [34,35]. Presently, the investigation and the presence of enteric viruses in water sources remains at a stalemate, with possible negative impacts on public health. Despite the widespread use of FIB as a tool to monitor water quality, outbreaks of waterborne diseases still occur all over the world, including the occurrence of water-related illnesses in water that meet the FIB standards [36,37]. Previous studies showed a lack of correlation between bacterial concentrations and viral presence [16,33,38]. The results from this study also corroborated this lack of correlation and showed that enteric viruses were detected at the six sample sites where the water met the standard level. The high prevalence of enteric viruses should be of concern, as they prove there is continuous fecal contamination in environmental water used for irrigation, fishing, consumption, and more. In fact, regardless of construction or character, enteric viruses demonstrate great difference with bacteria, with all kinds of environmental factors, which possibly contributes to the lack of association between the two. In addition, human enteric viruses can survive in the environment for a longer period of time than bacteria due to their greater environmental resistance. It is of great importance that water quality monitoring indicators should adequately reflect the presence of viruses. The impropriety of FIB in areas of viral presence could explain the outbreaks of diseases where the water met the microbiological standards. The detection of enteric viruses in fresh water in Poyang Lake emphasized the possibility of using enhanced method for detecting enteric viruses as an alternative indicator for water quality, the need for monitoring waterborne pathogens in fresh water, and the importance of finding contamination sources in order to protect the health of freshwater users. 

However, detection of enteric viruses at a low level in a large volume of environment water has been a technical challenge. This is also an obstacle when using enteric viruses as an alternative indicator in China. The analysis of enteric viruses in water generally requires water sample collection, water concentration, virus detection and identification. Due to the low concentration of enteric virus in water, the current methods are still unable to detect directly the enteric viruses without further processing of water concentration, therefore. The step of water concentration is crucial, as for the results of the analysis relies heavily on it. In this study, the water concentration of two methods are filtration based, Tianjin’s protocol used positive charge filter material and Hawaii’s protocol used negative charge filter material. The electrostatic charge of most viruses will be negative at the pH of environment water [39]. Electronegative filters rely on the manipulation of the water sample to cause a net positive surface charge of a viral particle. The advantages to most electropositive filters are that they are easy to use, with no preconditioning of the water samples required [40]. 

In this study, the two methods have their applicability and limitations. Sensitive and quantitative detection of human enteric viruses is typically achieved through quantitative RT-PCR (RT-qPCR) [41]. Tianjin’s protocol can get specific concentrations of enteric virus and assess the risks finally, but the preparatory work is arduous, the process time is lengthy, and requires precise instruments and skillful experimenters [22]. Hawaii’s protocol is time-saving and highly efficiency but obtains qualitative results. It does not reflect the actual virus load of the concentrated water samples and it is not possible to draw reliable conclusions on their infectivity which is of major concern for public health.

The results of the two methods detecting nucleic acid have statistical significance: The quantitative method is superior to the qualitative method, even though the qualitative method still can show the presence of enteric virus in Poyang Lake. 

A well-calibrated method for detecting enteric viruses should exhibit reliability, efficiency, simplicity, and suitability for the analysis of any kinds of water sample. Current methods need to focus not only on the detection of enteric viruses, but also the infectious nature of viruses in the future. 

## 5. Conclusions

The aim of this study was to demonstrate the feasibility of detecting enteric viruses in water as previously established in laboratory settings. In addition, the study found no correlation between bacterial indicators and enteric viruses in the environment, which supports the findings of prior studies. The quantitative method is superior to the qualitative method, even though the qualitative method still can show the presence of enteric virus in Poyang Lake. 

## Figures and Tables

**Table 1:** Sites sampled in the Poyang Lake.

Location	Details
1. Qingshan Zha	The southern branch of Gan River, Nanchang City
2. Guanniao Tai	Located in the Jiangxi Provincial Nature Reserve, in the southwestern region of Poyang Lake, Nanchang City
3. Tuo Shan	Located in the Jiangxi Provincial Nature Reserve, in the southwestern region of Poyang Lake, Nanchang City
4. Wu Cheng	In the north-central region of Poyang Lake, Jiujiang City
5. Xing Zi	In the north-central region of Jiangxi Province, Jiujiang City
6. Du Kou	At the junction of Poyang Lake and Yangtze River in Jiujiang City

**Table 2:** Quantitative PCR primer set and reaction condition ^a^.

Virus	Primer	Oligo Sequence (5′ → 3′)	Length (bp)	Reference
NoV II	NV-II-F	CARGARBCNATGTTYAGRTGGATGAG	98	[22]
	NV-II-R	TCGACGCCATCTTCATTCACA		
	NV-II-P	5′-(FAM)TGGGAGGGCGATCGCAATCT(TAMRA)-3’		
EoV	EV-F	GTGGCRGTGGCTGCGYT	204	[22]
	EV-R	ACCCAAAGTAGTCGGTTCCGC		
	EV-P	5′-(FAM)ATTAGCCGCATTCAGGGGCCGGA(TAMRA)-3’		
AdV	Adev-F	AACTTTCTCTCTTAATAGACGCCCC	87	[22]
	Adev-R	TGTCCACTAGTCCAAGAGGTGC		
	Adev-P	5′-(FAM)GCTGACACGGGCACTCTTCGC(TAMRA)-3’		

a. R = A + G, Y = C + T, B = C + G + T, N = A + T + C + G.

**Table 3:** Qualitative PCR primer set ^a^.

Virus	Primer	Oligo Sequence (5′ → 3′)	Length (bp)	Reference
NoV II	COG2F	CARGARBCNATGTTYAGRTGGATGAG	97	[20,30]
	COG2R	TCGACGCCATCTTCATTCACA		
EoV	EQ-1	ACATGGTGTGAAGAGTCTATTGAGCT	142	[28,31]
	EQ-2	CCAAAGTAGTCGGTTCCGC		
AdV	ADV-F	GCCACGGTGGGGTTTCTAAACTT	132	[20,21,32]
	ADV-R	GCCCCAGTGGTCTTACATGCACATC		

a. R = A + G, Y = C + T, B = C + G + T, N = A + T + C + G.

**Table 4:** Optimized amplification conditions and detection limits of successful primer sets.

Virus	Primer Set	T_A_^a^	[MgCl_2_]	[Primer]	BSA ^b^	Detection Limit ^c^	Reference
NoV II	COG2F/COG2R	52~60 °C	2 mM	800 nM	+	10^−6^–10^−7^x	[20,30]
EoV	EQ-1/EQ-2	58~60 °C	1.5 mM	600 nM	+	10^−7^x	[28,31]
AdV	ADV-F/ADV-R	60 °C	1.5 mM	600 nM	+	10^−6^–10^−7^x	[20,21,32]

a: T_A_ = Annealing temperature. b: At final concentration of 0.1 mg/mL þ: Addition of BSA improved amplification signal. c: The detection limits were based on the highest dilution giving a clear positive signal after RT-PCR.

**Table 5:** Concentration of bacterial indicator in Poyang Lake among different seasons and sampling sites (CFU/mL).

Title	Group	N	ACC	TC	FC	*E. coli*	ENT
Seasons	Summer	12	1.45 × 10^3^	0	0	0	4.00
	Autumn	12	1.28 × 10^3^	5.00 × 10^1^	5.00 × 10^1^	0	0
	Winter	12	3.15 × 10^2^	0	0	0	2.50 × 10^1^
Flood seasons	Rainy/wet season	12	1.45 × 10^3^	0	0	0	4.00
	Dry season	24	3.90 × 10^2^	2.50 × 10^1^	0	0	0
Sampling sites	1. Qingshan zha	6	3.00 × 10^3^	4.00 × 10^2^	0	1.0 × 10^2^	4.75 × 10
	2. Guanniao Tai	6	1.30 × 10^2^	0	0	0	0
	3. Tuoshan	6	5.25 × 10^2^	2.50 × 10^1^	9.25 × 10	0	0
	4. Wucheng	6	1.75 × 10^3^	0	0	0	0
	5. Xingzi	6	3.68 × 10^2^	2.50 × 10^1^	2.50 × 10^1^	0	5.00
	6. Dukou	6	3.10 × 10^4^	2.50 × 10^1^	2.50	0	7.00

**Table 6:** Detection of enteric viruses among different seasons and sampling sites through Hawaii’s method qualitative PCR.

	Group	N	NoV II	ENV	AdV
Seasons	Summer	12	2 (16.67)	9 (75.00)	9 (75.00)
	Autumn	12	7 (58.33)	5 (41.67)	7 (58.33)
	Winter	12	4 (33.33)	8 (66.67)	8 (66.67)
Flood seasons	Rainy/wet season	12	2 (16.67)	9 (75.00)	9 (75.00)
	Dry season	24	11 (45.83)	13 (54.17)	15 (62.50)
Sampling sites	1. Qingshan zha	6	2 (33.33)	2 (33.33)	2 (33.33)
	2. Guanniao Tai	6	3 (50.00)	3 (50.00)	3 (50.00)
	3. Tuoshan	6	1 (16.67)	3 (50.00)	3 (50.00)
	4. Wucheng	6	3 (50.00)	4 (66.67)	6 (100.00)
	5. Xingzi	6	1 (16.67)	5 (83.33)	5 (83.33)
	6. Dukou	6	3 (50.00)	5 (83.33)	5 (83.33)
Total		36	13 (36.11)	22 (61.11)	24 (66.67)

**Table 7:** Detection of enteric viruses among different seasons and sampling site through Tianjin’s method quantitative PCR (GC/L) ^a^.

Time (Year/Month)	Site	NoV II	ENV	AdV
2016/05	1. Qingshan zha	9194	3,307,965	91,357
	2. Guanniao Tai	1305	6678	18,724
	3. Tuoshan	0	567,784	39,011
	4. Wucheng	8488	156,782	68,253
	5. Xingzi	453,797	229,082	575,728
	6. Dukou	4,159,765	456,673	598,343
2016/06	1. Qingshan zha	9,877,789	2906,864	668,8789
	2. Guanniao Tai	0	6897	458,765
	3. Tuoshan	0	68,079	105,5445
	4. Wucheng	9100	25,634	897,790
	5. Xingzi	567,734	34,457	8,779,980
	6. Dukou	2,345,900	455,648	899,878
2016/10	1. Qingshan zha	401,920	386,1981	1,050,365
	2. Guanniao Tai	100,295	7,468,484	556,939
	3. Tuoshan	0	0	0
	4. Wucheng	38,981,076	1,673,658	573,600
	5. Xingzi	22,524,039	3,300,644	789,726
	6. Dukou	183,514	2,293,564	428,490
2016/11	1. Qingshan zha	1,257,891	2,837,055	2,000,810
	2. Guanniao Tai	113,051	6,330,130	718,724
	3. Tuoshan	56,680,339	5,741,566	968,012
	4. Wucheng	857,488	1,391,322	558,253
	5. Xingzi	55,374,097	2,027,416	755,727
	6. Dukou	6,154,197,581	955,726	698,343
2016/12	1. Qingshan zha	102,608,660	538,5941	2,461,629
	2. Guanniao Tai	0	0	0
	3. Tuoshan	4,935,579	8,003,342	631,161
	4. Wucheng	0	0	1220,268
	5. Xingzi	13,252,818	1,124,448	313,665
	6. Dukou	743,226	801,767	164,452
2017/01	1. Qingshan zha	5869	0	721,281
	2. Guanniao Tai	0	678,880	721,281
	3. Tuoshan	0	0	765,789
	4. Wucheng	7899	778,891	0
	5. Xingzi	10,100	898,761	8,793,346
	6. Dukou	3,567,824	700,876	808,803

a. GC is genome copies.

**Table 8:** Detection of enteric viruses among different seasons and sampling site through Tianjin’s method.

	Group	N	NoV II	ENV	AdV
Season	Summer	12	9 (75.00)	12 (100.00)	12 (100.00)
	Autumn	12	11 (91.67)	11 (91.67)	11 (91.67)
	Winter	12	8 (66.67)	8 (66.67)	10 (83.33)
Flood season	Rainy/wet season	12	9 (75.00)	12 (100.00)	12 (100.00)
	Dry season	24	19 (79.17)	19 (79.17)	21 (87.50)
Sampling site	1. Qingshan zha	6	6 (100.00)	6 (100.00)	6 (100.00)
	2. Guanniao Tai	6	3 (50.00)	5 (83.33)	5 (83.33)
	3. Tuoshan	6	2 (33.33)	4 (66.67)	5 (83.33)
	4. Wucheng	6	5 (83.33)	5 (83.33)	5 (83.33)
	5. Xingzi	6	6 (100.00)	6 (100.00)	6 (100.00)
	6. Dukou	6	6 (100.00)	6 (100.00)	6 (100.00)
Total		36	28 (77.78)	31 (86.11)	33 (91.67)

**Table 9:** Comparison the detection of enteric viruses through Tianjin’s method and Hawaii’s method by McNemar Test.

Hawaii’s Method	Tianjin’s Method			*p*
	Negative	Positive	Total	
NoV II				<0.001
Negative	6	17	23	
Positive	2	11	13	
Total	8	28	36	
ENV				0.035
Negative	2	12	14	
Positive	3	19	22	
Total	5	31	36	
AdV				0.035
Negative	0	12	12	
Positive	3	21	24	
Total	3	33	36	

**Table 10:** Comparison of Tianjin’s method and Hawaii’s method for viral concentration.

Difference between Two Methods	Tianjin’s Method	Hawaii’s Method
1. Volume of water sample used	50 L	500–2000 mL
2. Final volume of eluent	40 mL	<5 mL
3. Viral concentration time	≥6 h	≤1 h
4. Starting volume of nucleic acid	1 mL for DNA; 140 µL for RNA;	≤600 µL for both DNA and RNA
5. Final volume of nucleic acid	100 µL for DNA; 80 µL for RNA;	15 µL for DNA; 45 µL for RNA;
6. Kit price (Quantity: 50 Preps)	QIAamp Viral RNA Mini Kit: ¥3010 ($477.38/€390.20/£339.94)	The Power Water^®^ RNA Isolation Kit: ¥4500 ($713.69/€583.36/£508.22)
	UNIQ-10 Order Viral Genome DNA Extraction Kit: ¥544 ($86.28/€70.52/£61.44)	

**Table 11:** Correlation of enteric viruses between bacteriological index in Poyang Lake.

Enteric Virus	Spearman’s Correlation Coefficients (*p*-Value Is Two-Tailed)				
	ACC	TC	FC	*E. coli*	*ENT*
NV II	−0.055 (0.731)	−0.084 (0.595)	−0.162 (0.304)	−0.106 (0.506)	0.083 (0.629)
EoV	0.173 (0.273)	0.03 5(0.828)	−0.131 (0.409)	−0.034 (0.830)	0.076 (0.660)
AdV	0.160 (0.312)	0.075 (0.635)	0.002 (0.988)	−0.176 (0.265)	0.111 (0.520)

## Abbreviations

NoV GII	Norovirus geno-groups II
Eov	Enterovirus
Adv	Adenovirus
FIB	Fecal indicator bacteria
TC	Total coliform
FC	Fecal coliform
HCl	Hydrochloric acid
ACC	Aerobic bacterial count
PCR	Polymerase chain reaction
RT-PCR	Reverse transcript-polymerase chain reaction
BLAST	Basic local alignment search tool
EPA	Environmental protection agency
